# High-Throughput Strategies for the Discovery of Anticancer Drugs by Targeting Transcriptional Reprogramming

**DOI:** 10.3389/fonc.2021.762023

**Published:** 2021-10-01

**Authors:** Lijun Huang, Xiaohong Yi, Xiankuo Yu, Yumei Wang, Chen Zhang, Lixia Qin, Dale Guo, Shiyi Zhou, Guanbin Zhang, Yun Deng, Xilinqiqige Bao, Dong Wang

**Affiliations:** ^1^ State Key Laboratory of Southwestern Chinese Medicine Resources, School of Basic Medical Sciences, Chengdu University of Traditional Chinese Medicine, Chengdu, China; ^2^ School of Pharmacy, Chengdu University of Traditional Chinese Medicine, Chengdu, China; ^3^ Department of Infectious Diseases, 404 Hospital of Mianyang, Mianyang, China; ^4^ National Engineering Research Center for Beijing Biochip Technology, Beijing, China; ^5^ Medical Innovation Center for Nationalities, Inner Mongolia Medical University, Hohhot, China

**Keywords:** transcriptional reprogramming, anticancer drug discovery, high-throughput screening, L1000, HTS^2^

## Abstract

Transcriptional reprogramming contributes to the progression and recurrence of cancer. However, the poorly elucidated mechanisms of transcriptional reprogramming in tumors make the development of effective drugs difficult, and gene expression signature is helpful for connecting genetic information and pharmacologic treatment. So far, there are two gene-expression signature-based high-throughput drug discovery approaches: L1000, which measures the mRNA transcript abundance of 978 “landmark” genes, and high-throughput sequencing-based high-throughput screening (HTS^2^); they are suitable for anticancer drug discovery by targeting transcriptional reprogramming. L1000 uses ligation-mediated amplification and hybridization to Luminex beads and highlights gene expression changes by detecting bead colors and fluorescence intensity of phycoerythrin signal. HTS^2^ takes advantage of RNA-mediated oligonucleotide annealing, selection, and ligation, high throughput sequencing, to quantify gene expression changes by directly measuring gene sequences. This article summarizes technological principles and applications of L1000 and HTS^2^, and discusses their advantages and limitations in anticancer drug discovery.

## Introduction

Transcriptional reprogramming is a cause of cancer progression and recurrence. Gurdon first confirmed that differentiated somatic cells were plastic in nature and are reprogrammable into other cell fates (1). A cancer cell may present multiple phenotypes by reprogramming and changing its identity, inducing heterogeneity among tumor cells (2). Tumor heterogeneity is the major cause of drug resistance in cancer. The cancer stem cell (CSC) model and the clonal evolution model can be used to explain tumor heterogeneity. It was proposed that CSCs are derived from genetically and epigenetically altered stem cells or progenitor cells and possess self-renewal potential to sustain tumor mass, immune escape and drug resistance (3, 4). The clonal evolution model results from the inherent genomic instability of cancer cells, leading to genetic and epigenetic changes (4). The epigenetic changes, such as DNA methylation and histone acetylation, are vital for cancer progress (5). It is clear that transcriptional reprogramming involves almost all of these regulations.

Transcriptional reprogramming drives the diversification of tumor cells and causes tumor deterioration, such as hyperproliferation, invasion, metastasis, immune evasion, and drug resistance, and eventually causes cancer progression and recurrence. Hence, transcriptional reprogramming has emerged as a promising drug target for cancer therapy.

The detailed regulation mechanisms of transcriptional reprogramming are still poorly understood, and this makes effective drug discovery against this process difficult. Genomic instability (6), transcriptional factors (7–9), DNA methylation of tumor suppressor genes (10), unbalanced histone modifications (11, 12), aberrant Wnt signal pathway (13), PI3K signaling (14), TGF-β, and Erk/MAPK signaling (15) have been reported as some reasons for cell reprogramming and malignant transformation. Wang et al. established a principle for cell type-specific transcriptional reprogramming: Cell type-specific factors coupled with general transcriptional factors, which form a new cell-specific enhancer network, that other regulated factors can activate, and this may promote tumor cell progression (16). However, these discoveries explain only a limited part of transcriptional reprogramming. Thus, further elucidation of the transcriptional reprogramming mechanisms in normal and cancer cells may help develop cancer therapy strategies.

The gene expression signature might be a suitable readout for high-throughput drug discovery targeting transcriptional reprogramming. The expression changes of a group of interesting genes occur as a result of transcriptional reprogramming. This review, summarizes two published gene-expression signature-based high-throughput drug discovery strategies targeting transcriptional reprogramming: L1000 and high-throughput sequencing-based high-throughput screening (HTS^2^), introducing their technological principle and discussing their applications in drug discovery.

## L1000 as a Luminex Bead-Based High-Throughput Screening Strategy

L1000 is used to generate the next generation Connectivity Map (CMap) with higher throughput (17). CMap, which connects small molecules, genes, and diseases through gene signature, was first piloted in 2006 (18). By treating MCF7, PC3, HL60, and SKMEL5 cells with 164 distinct compounds and analyzing mRNA expression using Affymetrix microarrays (18), 564 datasets were generated. The small-scale datasets of pilot CMap limit its use as a powerful resource. Therefore, a low-cost approach, L1000, was proposed to produce large-scale gene signatures through a reduced representation of transcriptome (17).

**Figure 1:**
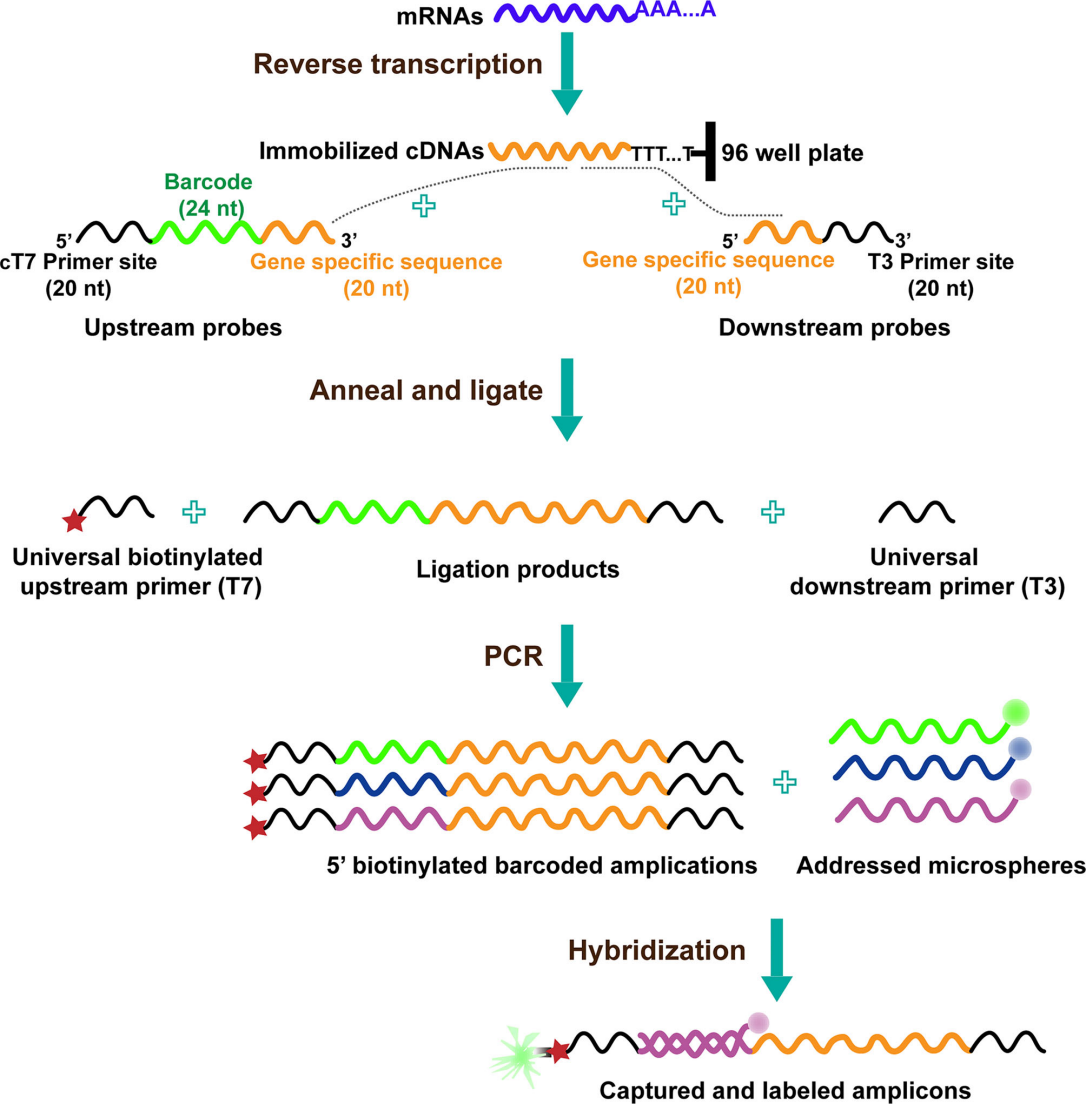
The diagram of L1000 technology (19).

The procedure of L1000 technology includes the following steps (17): cells treated with distinct perturbations in 384-well plates are lysed, and their mRNAs are captured on oligo-dT-coated plates after which it is reverse-transcribed to cDNA. The oligonucleotide probe comprises locus-specific sequences, 24-mer unique barcode sequences, and universal primer sequence sites. Then, the oligonucleotide probes are annealed to cDNA, and the juxtaposed upstream and downstream probe pairs are ligated; the upstream probe consists of a unique barcode sequence. After the above process, the ligations are used as a template and subjected to PCR amplification; using the universal 5′ biotinylated T7 primer and T3 primer pairs, the final amplicons are gene-specific, barcoded, and biotinylated. After that, each barcode of the amplification product hybridizes to polystyrene microsphere (bead with fluorescence color) by complementary pairing, and the bead is stained with Streptavidin R-phycoerythrin conjugate. Because beads are available in a maximum of 500 colors, two transcripts are hybridized with the same bead color. Finally, the hybridized beads coupling to barcodes are detected and analyzed using Luminex FlexMap 3D flow cytometer. The colors of beads indicate gene identity, whereas the fluorescence intensity of the phycoerythrin signal refers to gene abundance (**Figure 1**).

### The Application of L1000 in Cancer Drug Discovery

L1000 was used in discovering synergistic anti-glioblastoma drugs. Glioblastoma is a type of fatal brain cancer, containing highly heterogeneous cell populations. These cell populations have various of gene signatures; therefore, both radiation and chemotherapy for glioblastoma often induce inherent or acquired resistance (20). To overcome resistance, combination therapies are considered. Therefore, glioblastoma patient-specific genes, analyzed using TCGA database and L1000 transcriptional profiling data, were used to predict drugs that produce a synergistic combination against glioblastoma. Because of this, the combinations of GSK-1070916 with JQ1, alisertib with JQ1, gemcitabine with mitoxantrone, and gemcitabine with imatinib were predicted and verified to be synergistic in inhibiting glioblastoma (21).

L1000 was applied in finding a drug against renal cell carcinoma (RCC). *DDX3X* is involved in RNA metabolism (22–24). *DDX3X* is epigenetically downregulated in RCC (25). According to transcriptomic analysis, lower levels of *DDX3X* promote gene expression in the SPINK1-metallothionein pathway, leading to tumor growth, metastasis, and poorer prognosis of RCC patients (25, 26). Based on the *DDX3X* gene signature and L1000 datasets, digoxin was identified to reverse the gene signature generated by low *DDX3X*, thus inhibiting cell proliferation and metastasis (25).

Some FDA-approved drugs could be repurposed using gene-expression signature and L1000 datasets. *HMGA2* encodes a chromatin protein that promotes tumor progression and poor treatment (27–30). To discover a specific inhibitor that targets HMGA2, the combination of L1000 platform and GEO database were analyzed. According to the analysis, the approved antifungal drug ciclopirox is a novel potential inhibitor that targets HMGA2, and the molecular docking results further showed that ciclopirox directly interacts with the AT-hook motif of HMGA2. The functional assays also showed that ciclopirox represses colorectal cancer cell growth by inducing cell cycle arrest and apoptosis (31).

L1000 was applied to discover drugs against quiescent spheroids. Cells residing within the center of solid tumors lack nutrients and oxygen, and most of these cells are transcriptionally reprogrammable, quiescent, and negative to antiproliferation therapy (32). Senkowski et al. used L1000 technology to generate gene-expression signatures from monolayer cultures and tumor cell spheroids treated with 22 drugs. Spheroids were cultured in fresh culture medium or media that were similar to hypoxic tumor parenchyma. According to the analysis of L1000 expression profiles, the mevalonate pathway is upregulated as a result of oxidative phosphorylation (OXPHOS) inhibition in quiescent cells. Thus, this study indicated that the application of OXPHOS inhibitors (such as salinomycin, nitazoxanide and antimycin A) and mevalonate pathway inhibitors (such as simvastatin) synergistically inhibits quiescent spheroids (32).

### Pros and Cons of L1000

L1000 establishment of the causality among drug, gene, and disease provides the mechanism of action of compounds or gene perturbations, and possesses the ability to predict the function or possible side effects of compounds systemically (17). L1000 can detect the expression of as many as 1,000 genes simultaneously. DNA oligos for about 1,000 genes are designed and amplified, the biotin-labeled amplicons are then hybridized to Luminex beads. The beads’ colors and the fluorescence signals attached to beads are detected. Based on hybridization, L1000 is capable of expanding the signal of non-abundant transcripts and measuring their expression (17).

L1000 is inexpensive, rapid, and flexible when used to profile gene expression on a large scale (19, 33). L1000 uses ligation-mediated amplification to measure gene expressions, which uses 40nt gene-specific sequences for transcriptome detection other than full-length transcriptome sequencing. Therefore, compared to RNA-seq technology, the reduced representation of transcriptome makes L1000 a cost-effective method for gene expression profiling. Furthermore, several datasets generated by L1000 have been published. Due to the above advantages, it has generated 1,319,138 profiles from 42,080 perturbations on nine cell types and covered 473,647 signatures (17). These datasets are open to the public and should be helpful for researchers seeking to discover drugs against cancer and other human diseases.

However, there are also limitations for L1000. First, only 1,000 genes can be detected. Only 500 bead colors are commercially available, and thus, a maximum of 500 genes (one gene/one color) can be generally identified. Although L1000 allows the detection of two transcripts by a single bead color, which doubles the gene number of identified genes, still the number of genes detected cannot be more than 1,000 (17). Besides, L1000 assay uses polystyrene beads. Although polystyrene beads are the first generation of beads for Luminex assays, their accuracy and precision are reduced, accompanied by leaking and clogging during the protocol of washing in the plates (34).

The protocol of L1000 is complicated. Before beginning L1000 assay, 1000 pairs of gene-specific sequences and 1,000 barcode sequences need to be designed, and Luminex beads need to be joined with 500 barcodes. After preparation for work, the cells are lysed into mRNA, and the mRNA needs to be attached to the oligo-dT plate. After that, mRNA is reverse-transcribed into cDNA. The cDNA servers as a template to combine the specific gene sequences labeled with barcodes, and the upstream and downstream specific gene sequences were ligated using the T4 ligase. Then, the ligations are used as a transcriptional template for amplification using universal primers combined with biotin. Then, the amplicons are hybridized into beads and then phycoerythrin-labeled streptavidin. Finally, the bead’s color (gene identity) and the phycoerythrin signal (gene abundance) are detected. The technical characteristics of L1000 are summarized in **Table 1**.

**Table 1:** Comparation between L1000 and HTS^2^.

Technology	L1000	HTS^2^
Detection method	Luminex beads	High throughput sequencing
Gene detection	Indirect	Direct
Throughput	High	High
Detectable gene number	≤1000	Unlimited
Degree of automation	Low	High
Technological Upgrade Potential	Low	High
Number of published datasets	Large	Small

## HTS^2^: High-Throughput Sequencing-Based High-Throughput Screening

**Figure 2:**
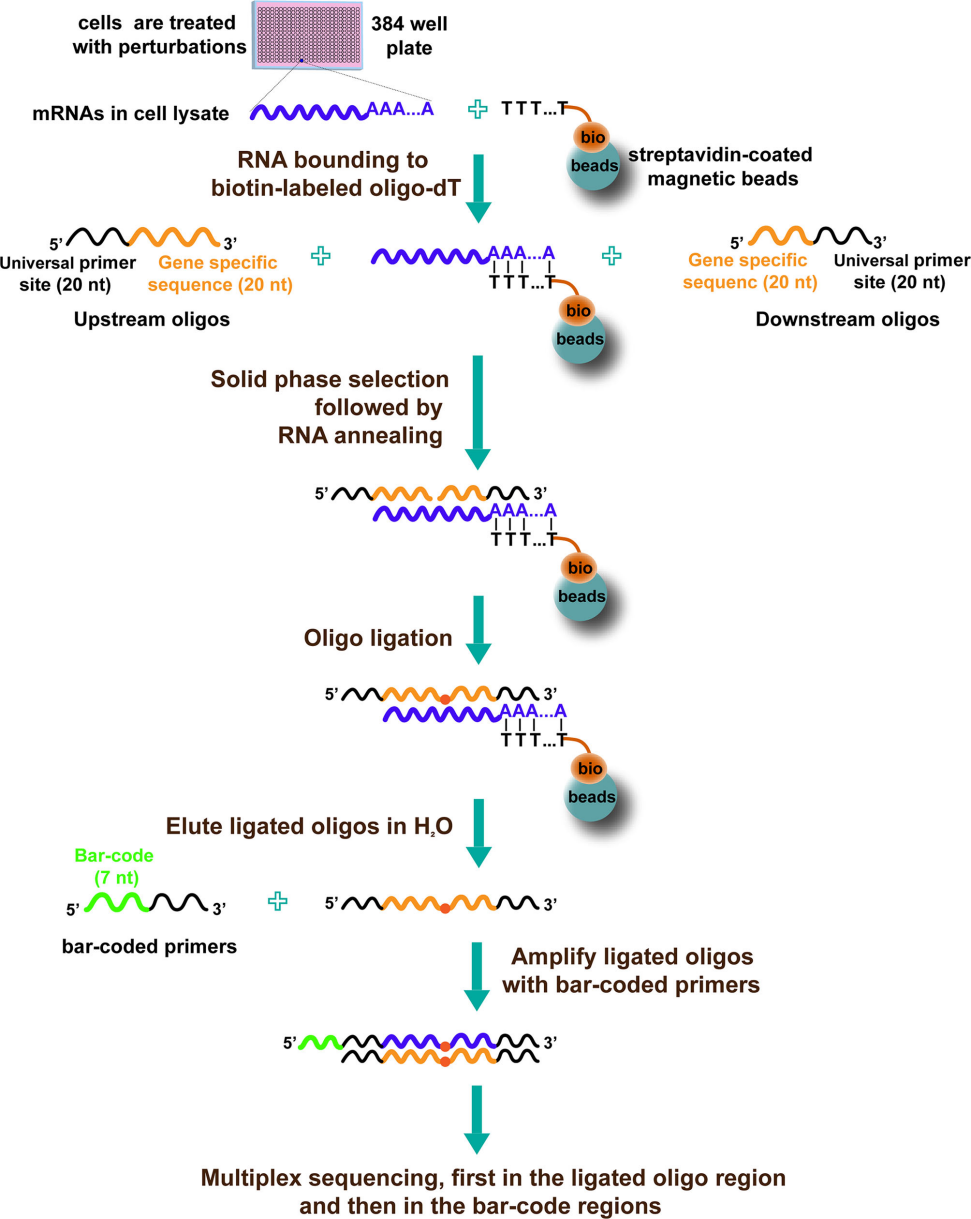
The diagram of HTS^2^ (35).

Another high-throughput approach to discover drugs by targeting transcriptional reprogramming is HTS^2^ (35). The procedure of HTS^2^ is as follows (**Figure 2**): cells are treated with various perturbations in 384-well plates. Then, cells are lysed, and the mRNA in the lysate is bound to biotin-labeled oligo-dT, joined with streptavidin-coated magnetic beads. After that, upstream oligos (consisting of 5′ universal primer site and 20nt gene-specific sequences) and downstream oligos (containing another 20nt gene-specific sequences adjacent to upstream and 3′ universal primer site) are annealed to mRNA template and ligated with T4 ligase. The ligated products with 40nt gene-specific sequences are used as templates and subjected to PCR amplification. The PCR primers contain a barcode site, which identifies samples; different genes from the same sample share the same barcode. Finally, the amplicons, including barcode and 40nt ligated oligo regions, are sequenced using next-generation sequencing technology (35).

### The Application of HTS^2^ in Cancer Drug Discovery by Targeting Transcriptional Reprogramming

HTS^2^ technology is suitable for pathway-centric discovery of anticancer drugs. Androgen receptor (AR) overexpression may lead to androgen resistance and the development of incurable prostate cancer (36). HTS^2^ was applied to identify drugs that block the expression of signature genes regulated by AR in prostate cancer cells, which indicates that this candidate drug may inhibit the AR pathway. According to this study, cardiac glycosides block the expression of AR target genes and inhibits the proliferation of androgen-sensitive and androgen-resistant prostate cancer cells by causing AR destabilization (35).

HTS^2^ facilitates the discovery of anti-metastasis drugs. Tumor metastasis is the movement of tumor cells from a primary site to distant organs that they progressively colonize (8). Tumor metastasis is the cause of death for 90% of cancer patients, and no currently available therapies target this multi-step process.

Metastasis may be regulated by transcriptional reprogramming. It was reported that the transcription factor *FOXA1* is upregulated and drives the transcriptional reprogramming to promote pancreatic ductal adenocarcinoma cell metastasis (8). Teng et al. discovered that liver metastatic colorectal cancer (CRC) cells acquire higher expression of liver-specific genes than primary colorectal tumor cells. This transcriptional reprogramming is driven by liver-specific *FOXA2* and *HNF1A*, which can bind to reshaped enhancers of liver metastatic CRC cells, and promote CRC liver metastasis (9).

Gene-expression signatures are used to characterize cancer metastasis (37–39). To effectively discover drugs against breast cancer lung metastasis (BCLM), HTS^2^ technology and BCLM-associated gene signature were combined and analyzed. It was found that ponatinib represses the expression of BCLM signature genes through the inhibition of *JUN* transcription and degradation of the c-Jun protein, ultimately inhibiting BCLM (40).

HTS^2^ can also be used to explore the mechanisms of action of anticancer herbs. Combined with network pharmacology, HTS^2^ was used to unveil the biological basis of medicine with complex ingredients, such as traditional Chinese medicine (TCM) in cancer therapy (41, 42) as well as other diseases (43). Zheng et al. utilized HTS^2^ to measure the function of 166 compounds derived from TCM on 420 antitumor or immune-related genes. The results from gene signature showed that compounds from health-strengthening herbs increase immune effects in tumor immune microenvironment and tumor prevention (41). Guizhi Fuling Decoction (GFD) is a classic TCM prescription used in treating gynecological tumors with an unclear mechanism. Dai et al. applied HTS^2^ technology along with systemic pharmacology to clarify the mechanisms of GFD in treating breast cancer; this revealed that GFD represses breast cancer through the inhibition of PI3K and MAPK signaling pathways (42).

HTS^2^ contributes to the discovery of combination immunotherapy agents against triple-negative breast cancer (TNBC). Low objective response rates (ORRs) of solid tumors create immune checkpoint blockade therapy failure in some aggressive cancers (44–46). ORRs are associated with tumor immunological phenotype (TIP) that leads to the extent of immune cell infiltration (47). Hot tumor is feasibly infiltrated by immune cells, which are regulated by tumor genes, including T helper1 (TH_1_)-type chemokines. Conversely, cold tumors are those that are not infiltrated by immune cells or are immune-ignorant (48). Due to epigenetic alteration and transcriptional reprogramming, hot tumor may be converted to cold tumor, leading to tumor immunosuppression (49). Small molecules can also epigenetically convert cold tumors to hot tumors by altering the gene expression of TH_1_ chemokines (50). To increase the ORRs of checkpoint blockade immunotherapy, combination targets or compounds are desirable. Wang et al. first determined the difference in TIP gene signature between cold and hot tumors. Combined with this gene signature, HTS^2^ technology was applied for the identification of immunotherapy combination agents in TNBC. The results showed that aurora kinase inhibitors reprogram the expression of TIP gene signature and thus promote effective T-cell infiltration into the tumor microenvironment, significantly improving anti-programmed cell death 1 (PD-1) efficacy in preclinical models (51).

### Pros and Cons of HTS^2^

First, HTS^2^ can detect unlimited genes. It was reported that the expression of >3,000 genes was directly examined in one reaction by HTS^2^ (52). In principle, all human genes (~22,000 genes) can be detected by HTS^2^ in one reaction, since it takes advantage of the powerful high-throughput sequencing technologies. More importantly, these high-throughput sequencing technologies should be developed and improved quickly, to increase the detection capability of HTS^2^ in the future.

Second, HTS^2^ directly detects gene expression. HTS^2^ detects gene signatures using high-throughput sequencing technology, detecting and quantifying gene expression by reading out their sequence directly. Due to this, the possibility of misreading should be rare. Third, the experimental scheme of HTS^2^ is fully amenable to direct transcript analysis in cell lysate and automation, which are two critical parameters for high-throughput applications. The annealing step of HTS^2^ is fully compatible in cell lysis containing detergent and high salt. After it is captured by streptavidin-coated magnetic beads, all subsequent washing and ligation steps are conducted on the solid phase. Furthermore, this HTS^2^ strategy can be fully implemented on an automated robot (35).

However, there are also some challenges for HTS^2^ strategy. First, even though HTS^2^ could detect the expression of unlimited genes in principle, the number of detected genes reported so far is no more than 4,000. It would be much better if full transcriptome could be examined in one reaction using HTS^2^ in the future. Alternatively, there are only few pieces of literature, which applied this technology, that have been were published so far; more studies need to be published to demonstrate the broad utility of the HTS^2^ technology in both basic and translational research. The technical characteristics of HTS^2^ are shown in **Table 1**.

## Conclusions

Transcriptional reprogramming is involved in cancer initiation, progression, and metastasis; thus, it is a potential target for anticancer drug development. L1000 and HTS^2^ are gene-expression signature-based high-throughput approaches, suitable for drug discoveries targeting transcriptional reprogramming. Notably, all these two technologies are based on bulk RNA. Recently, the gene expression changes in single cells are making significant impact on the understanding of almost all the processes of life. Meanwhile, single cell RNA sequencing was also reported facilitating drug discovery (53–55). So far, these technologies of single cell RNA sequencing are limited by high cost or low multiplex for the application in the high throughput drug discovery. However, the measurement of gene expression changes in single cells represents another potential high-throughput approach for the drug discovery to target transcriptional reprogramming. Both of them show advantages as well as limitations. Undoubtedly, these two technologies offer powerful and effective platforms for large-scale genetics and chemical genetics studies, and anticancer drug discovery.

## Author Contributions

DW and XB designed and supervised the article. LH summarized literatures about transcriptional reprogramming, L1000 and HTS^2^ technology, and drafted the manuscript. XHY, XKY, YW, CZ, LQ, DG, SZ, GZ and YD contribute to the writing of this manuscript. All authors contributed to the article and approved the submitted version.

## Funding

This work was supported by the National Natural Science Foundation of China (81673460), Key Projects of Science and Technology Plan of Inner Mongolia Autonomous Region (201802115), Sichuan Youth Science and Technology Innovation Research Team of Experimental Formulology (2020JDTD0022), and “Xinglin Scholars” scientific research promotion plan of Chengdu University of Traditional Chinese Medicine (BSH2019017).

## Conflict of Interest

The authors declare that the research was conducted in the absence of any commercial or financial relationships that could be construed as a potential conflict of interest.

## Publisher’s Note

All claims expressed in this article are solely those of the authors and do not necessarily represent those of their affiliated organizations, or those of the publisher, the editors and the reviewers. Any product that may be evaluated in this article, or claim that may be made by its manufacturer, is not guaranteed or endorsed by the publisher.

## References

[1] Gurdon JB Elsdale TR Fischberg M. Sexually Mature Individuals of Xenopus Laevis From the Transplantation of Single Somatic Nuclei. Nature (1958) 182(4627):64–5. doi: 10.1038/182064a0 13566187

[2] Hass R von der Ohe J Ungefroren H. The Intimate Relationship Among EMT, MET and TME: A T(ransdifferentiation) E(nhancing) M(ix) to Be Exploited for Therapeutic Purposes. Cancers (Basel) (2020) 12(12):3674. doi: 10.3390/cancers12123674 PMC7762343 33297508

[3] Saito S Lin YC Nakamura Y Eckner R Wuputra K Kuo KK. Potential Application of Cell Reprogramming Techniques for Cancer Research. Cell Mol Life Sci (2019) 76(1):45–65. doi: 10.1007/s00018-018-2924-7 30283976 PMC6326983

[4] Welch DR. Tumor Heterogeneity–A 'Contemporary Concept' Founded on Historical Insights and Predictions. Cancer Res (2016) 76(1):4–6. doi: 10.1158/0008-5472.CAN-15-3024 26729788 PMC4773023

[5] Gong LQ Yan Q Zhang Y Fang XN Liu BL Guan XY. Cancer Cell Reprogramming: A Promising Therapy Converting Malignancy to Benignity. Cancer Commun (Lond) (2019) 39(1):48. doi: 10.1186/s40880-019-0393-5 31464654 PMC6716904

[6] Negrini S Gorgoulis VG Halazonetis TD. Genomic Instability–an Evolving Hallmark of Cancer. Nat Rev Mol Cell Biol (2010) 11(3):220–8. doi: 10.1038/nrm2858 20177397

[7] Denny SK Yang D Chuang CH Brady JJ Lim JS Gruner BM. Nfib Promotes Metastasis Through a Widespread Increase in Chromatin Accessibility. Cell (2016) 166(2):328–42. doi: 10.1016/j.cell.2016.05.052 PMC5004630 27374332

[8] Roe JS Hwang CI Somerville TDD Milazzo JP Lee EJ Da Silva B. Enhancer Reprogramming Promotes Pancreatic Cancer Metastasis. Cell (2017) 170(5):875–88.e20. doi: 10.1016/j.cell.2017.07.007 28757253 PMC5726277

[9] Teng SS Li YE Yang M Qi R Huang YM Wang QY. Tissue-Specific Transcription Reprogramming Promotes Liver Metastasis of Colorectal Cancer. Cell Res (2020) 30(1):34–49. doi: 10.1038/s41422-019-0259-z 31811277 PMC6951341

[10] Jones PA Laird PW. Cancer Epigenetics Comes of Age. Nat Genet (1999) 21(2):163–7. doi: 10.1038/5947 9988266

[11] Hessmann E Johnsen SA Siveke JT Ellenrieder V. Epigenetic Treatment of Pancreatic Cancer: Is There a Therapeutic Perspective on the Horizon? Gut (2017) 66(1):168–79. doi: 10.1136/gutjnl-2016-312539 PMC5256386 27811314

[12] Schneider G Kramer OH Schmid RM Saur D. Acetylation as a Transcriptional Control Mechanism-HDACs and HATs in Pancreatic Ductal Adenocarcinoma. J Gastrointest Cancer (2011) 42(2):85–92. doi: 10.1007/s12029-011-9257-1 21271301

[13] Katoh M. Canonical and non-Canonical WNT Signaling in Cancer Stem Cells and Their Niches: Cellular Heterogeneity, Omics Reprogramming, Targeted Therapy and Tumor Plasticity (Review). Int J Oncol (2017) 51(5):1357–69. doi: 10.3892/ijo.2017.4129 PMC5642388 29048660

[14] Van Keymeulen A Lee MY Ousset M Brohee S Rorive S Giraddi RR. Reactivation of Multipotency by Oncogenic PIK3CA Induces Breast Tumour Heterogeneity. Nature (2015) 525(7567):119–23. doi: 10.1038/nature14665 26266985

[15] Zheng YW Nie YZ Taniguchi H. Cellular Reprogramming and Hepatocellular Carcinoma Development. World J Gastroenterol (2013) 19(47):8850–60. doi: 10.3748/wjg.v19.i47.8850 PMC3870535 24379607

[16] Wang D Garcia-Bassets I Benner C Li WB Su X Zhou YM. Reprogramming Transcription by Distinct Classes of Enhancers Functionally Defined by eRNA. Nature (2011) 474(7351):390–4. doi: 10.1038/nature10006 PMC3117022 21572438

[17] Subramanian A Narayan R Corsello SM Peck DD Natoli TE Lu XD. A Next Generation Connectivity Map: L1000 Platform and the First 1,000,000 Profiles. Cell (2017) 171(6):1437–52.e17. doi: 10.1016/j.cell.2017.10.049 29195078 PMC5990023

[18] Lamb J Crawford ED Peck D Modell JW Blat IC Wrobel MJ. The Connectivity Map: Using Gene-Expression Signatures to Connect Small Molecules, Genes, and Disease. Science (2006) 313(5795):1929–35. doi: 10.1126/science.1132939 17008526

[19] Peck D Crawford ED Ross KN Stegmaier K Golub TR Lamb J. A Method for High-Throughput Gene Expression Signature Analysis. Genome Biol (2006) 7(7):R61. doi: 10.1186/gb-2006-7-7-r61 16859521 PMC1779561

[20] Qazi MA Vora P Venugopal C Sidhu SS Moffat J Swanton C. Intratumoral Heterogeneity: Pathways to Treatment Resistance and Relapse in Human Glioblastoma. Ann Oncol (2017) 28(7):1448–56. doi: 10.1093/annonc/mdx169 28407030

[21] Stathias V Jermakowicz AM Maloof ME Forlin M Walters W Suter RK. Drug and Disease Signature Integration Identifies Synergistic Combinations in Glioblastoma. Nat Commun (2018) 9(1):5315. doi: 10.1038/s41467-018-07659-z 30552330 PMC6294341

[22] Zhou Z Licklider LJ Gygi SP Reed R. Comprehensive Proteomic Analysis of the Human Spliceosome. Nature (2002) 419(6903):182–5. doi: 10.1038/nature01031 12226669

[23] Chao CH Chen CM Cheng PL Shih JW Tsou AP Lee YH. DDX3, a DEAD Box RNA Helicase With Tumor Growth-Suppressive Property and Transcriptional Regulation Activity of the P21waf1/Cip1 Promoter, Is a Candidate Tumor Suppressor. Cancer Res (2006) 66(13):6579–88. doi: 10.1158/0008-5472.CAN-05-2415 16818630

[24] Yedavalli VSRK Neuveut C Chi YH Kleiman L Jeang KT. Requirement of DDX3 DEAD Box RNA Helicase for HIV-1 Rev-RRE Export Function. Cell (2004) 119(3):381–92. doi: 10.1016/j.cell.2004.09.029 15507209

[25] Lin TC. DDX3X Is Epigenetically Repressed in Renal Cell Carcinoma and Serves as a Prognostic Indicator and Therapeutic Target in Cancer Progression. Int J Mol Sci (2020) 21(8):2881. doi: 10.3390/ijms21082881 PMC7215876 32326089

[26] Lin TC. DDX3X Multifunctionally Modulates Tumor Progression and Serves as a Prognostic Indicator to Predict Cancer Outcomes. Int J Mol Sci (2019) 21(1):281. doi: 10.3390/ijms21010281 PMC6982152 31906196

[27] Zhang SZ Mo QP Wang XC. Oncological Role of HMGA2 (Review). Int J Oncol (2019) 55(4):775–88. doi: 10.3892/ijo.2019.4856 31432151

[28] Di Cello F Hillion J Hristov A Wood LJ Mukherjee M Schuldenfrei A. HMGA2 Participates in Transformation in Human Lung Cancer. Mol Cancer Res (2008) 6(5):743–50. doi: 10.1158/1541-7786.MCR-07-0095 PMC3086547 18505920

[29] Wang XC Liu XY Li AYJ Chen LR Lai LL Lin HH. Overexpression of HMGA2 Promotes Metastasis and Impacts Survival of Colorectal Cancers. Clin Cancer Res (2011) 17(8):2570–80. doi: 10.1158/1078-0432.CCR-10-2542 PMC3079060 21252160

[30] Califano D Pignata S Losito NS Ottaiano A Greggi S De Simone V. High HMGA2 Expression and High Body Mass Index Negatively Affect the Prognosis of Patients With Ovarian Cancer. J Cell Physiol (2014) 229(1):53–9. doi: 10.1002/jcp.24416 23765903

[31] Huang YM Cheng CH Pan SL Yang PM Lin DY Lee KH. Gene Expression Signature-Based Approach Identifies Antifungal Drug Ciclopirox As a Novel Inhibitor of HMGA2 in Colorectal Cancer. Biomolecules (2019) 9(11):688. doi: 10.3390/biom9110688 PMC6920845 31684108

[32] Senkowski W Jarvius M Rubin J Lengqvist J Gustafsson MG Nygren P. Large-Scale Gene Expression Profiling Platform for Identification of Context-Dependent Drug Responses in Multicellular Tumor Spheroids. Cell Chem Biol (2016) 23(11):1428–38. doi: 10.1016/j.chembiol.2016.09.013 27984028

[33] Duan QN Flynn C Niepel M Hafner M Muhlich JL Fernandez NF. LINCS Canvas Browser: Interactive Web App to Query, Browse and Interrogate LINCS L1000 Gene Expression Signatures. Nucleic Acids Res (2014) 42(Web Server issue):W449–60. doi: 10.1093/nar/gku476 PMC4086130 24906883

[34] Kupcova Skalnikova H Cizkova J Cervenka J Vodicka P. Advances in Proteomic Techniques for Cytokine Analysis: Focus on Melanoma Research. Int J Mol Sci (2017) 18(12):2697. doi: 10.3390/ijms18122697 PMC5751298 29236046

[35] Li HR Zhou HY Wang D Qiu JS Zhou Y Li XQ. Versatile Pathway-Centric Approach Based on High-Throughput Sequencing to Anticancer Drug Discovery. Proc Natl Acad Sci USA (2012) 109(12):4609–14. doi: 10.1073/pnas.1200305109 PMC3311343 22396588

[36] Chen CD Welsbie DS Tran C Baek SH Chen R Vessella R. Molecular Determinants of Resistance to Antiandrogen Therapy. Nat Med (2004) 10(1):33–9. doi: 10.1038/nm972 14702632

[37] Kang YB Siegel PM Shu WP Drobnjak M Kakonen SM Cordón-Cardo C. A Multigenic Program Mediating Breast Cancer Metastasis to Bone. Cancer Cell (2003) 3(6):537–49. doi: 10.1016/s1535-6108(03)00132-6 12842083

[38] Minn AJ Gupta GP Siegel PM Bos PD Shu WP Giri DD. Genes That Mediate Breast Cancer Metastasis to Lung. Nature (2005) 436(7050):518–24. doi: 10.1038/nature03799 PMC1283098 16049480

[39] Bos PD Zhang XH Nadal C Shu WP Gomis RR Nguyen DX. Genes That Mediate Breast Cancer Metastasis to the Brain. Nature (2009) 459(7249):1005–9. doi: 10.1038/nature08021 PMC2698953 19421193

[40] Shao W Li SS Li L Lin KQ Liu XH Wang HY. Chemical Genomics Reveals Inhibition of Breast Cancer Lung Metastasis by Ponatinib via C-Jun. Protein Cell (2019) 10(3):161–77. doi: 10.1007/s13238-018-0533-8 PMC6338618 29667003

[41] Zheng JH Wu M Wang HY Li SS Wang X Li Y. Network Pharmacology to Unveil the Biological Basis of Health-Strengthening Herbal Medicine in Cancer Treatment. Cancers (Basel) (2018) 10(11):461. doi: 10.3390/cancers10110461 PMC6266222 30469422

[42] Dai YF Qiang WJ Yu XK Cai SW Lin KQ Xie L. Guizhi Fuling Decoction Inhibiting the PI3K and MAPK Pathways in Breast Cancer Cells Revealed by HTS^2^ Technology and Systems Pharmacology. Comput Struct Biotechnol J (2020) 18:1121–36. doi: 10.1016/j.csbj.2020.05.004 PMC7260686 32489526

[43] Dai YF Qiang WJ Gui Y Tan X Pei TL Lin KQ. A Large-Scale Transcriptional Study Reveals Inhibition of COVID-19 Related Cytokine Storm by Traditional Chinese Medicines. Sci Bull (Beijing) (2021) 66(9):884–8. doi: 10.1016/j.scib.2021.01.005 PMC7803147 33457042

[44] Xu J Saklatvala R Mittal S Deshmukh S Procopio A. Recent Progress of Potentiating Immune Checkpoint Blockade With External Stimuli-An Industry Perspective. Adv Sci (Weinh) (2020) 7(8):1903394. doi: 10.1002/advs.201903394 32328428 PMC7175294

[45] Lu ZH Peng Z Liu C Wang ZH Wang YK Jiao X. Current Status and Future Perspective of Immunotherapy in Gastrointestinal Cancers. Innovation (2020) 1(2):100041. doi: 10.1016/j.xinn.2020.100041 PMC8454608 34557714

[46] Schnell A Bod L Madi A Kuchroo VK. The Yin and Yang of Co-Inhibitory Receptors: Toward Anti-Tumor Immunity Without Autoimmunity. Cell Res (2020) 30(4):285–99. doi: 10.1038/s41422-020-0277-x PMC7118128 31974523

[47] Nagarsheth N Wicha MS Zou WP. Chemokines in the Cancer Microenvironment and Their Relevance in Cancer Immunotherapy. Nat Rev Immunol (2017) 17(9):559–72. doi: 10.1038/nri.2017.49 PMC5731833 28555670

[48] Galon J Bruni D. Approaches to Treat Immune Hot, Altered and Cold Tumours With Combination Immunotherapies. Nat Rev Drug Discov (2019) 18(3):197–218. doi: 10.1038/s41573-018-0007-y 30610226

[49] Nagarsheth N Peng DJ Kryczek I Wu K Li W Zhao E. PRC2 Epigenetically Silences Th1-Type Chemokines to Suppress Effector T-Cell Trafficking in Colon Cancer. Cancer Res (2016) 76(2):275–82. doi: 10.1158/0008-5472.CAN-15-1938 PMC4715964 26567139

[50] Peng DJ Kryczek I Nagarsheth N Zhao LL Wei S Wang WM. Epigenetic Silencing of TH1-Type Chemokines Shapes Tumour Immunity and Immunotherapy. Nature (2015) 527(7577):249–53. doi: 10.1038/nature15520 PMC4779053 26503055

[51] Wang HY Li SS Wang QY Jin ZS Shao W Gao Y. Tumor Immunological Phenotype Signature-Based High-Throughput Screening for the Discovery of Combination Immunotherapy Compounds. Sci Adv (2021) 7(4):eabd7851. doi: 10.1126/sciadv.abd7851 33523948 PMC10964968

[52] Qiao LS Huang WT Zhang XL Guo HY Wang D Feng QS. Evaluation of the Immunomodulatory Effects of Anti-COVID-19 TCM Formulae by Multiple Virus-Related Pathways. Signal Transduct Target Ther (2021) 6(1):50. doi: 10.1038/s41392-021-00475-w 33542177 PMC7860167

[53] Lei YL Tang R Xu J Wang W Zhang B Liu J. Applications of Single-Cell Sequencing in Cancer Research: Progress and Perspectives. J Hematol Oncol (2021) 14(1):91. doi: 10.1186/s13045-021-01105-2 34108022 PMC8190846

[54] Srivatsan SR McFaline-Figueroa JL Ramani V Saunders L Cao JY Packer J. Massively Multiplex Chemical Transcriptomics at Single-Cell Resolution. Science (2020) 367(6473):45–51. doi: 10.1126/science.aax6234 31806696 PMC7289078

[55] Wu H Wang C Wu S. Single-Cell Sequencing for Drug Discovery and Drug Development. Curr Top Med Chem (2017) 17(15):1769–77. doi: 10.2174/1568026617666161116145358 27848892

